# A tribute to my supervisor Professor Zhengyi Wu

**DOI:** 10.1007/s13238-018-0565-0

**Published:** 2018-07-11

**Authors:** Zhekun Zhou

**Affiliations:** 1 Key Laboratory of Tropical Forest Ecology, Xishuangbanna Tropical Botanical Garden, Chinese Academy of Sciences, Menglun, Mengla, 666303 Yunnan China; 2 Key Laboratory for Plant Diversity and Biogeography of East Asia, Kunming Institute of Botany, Chinese Academy of Sciences, Kunming, 650201 China

I was saddened and quite shocked when I received the phone called informing me that Professor Zhengyi Wu had died. It was the evening of June 20th, 2013. I had just got off a plane from Tibet and turned on my mobile phone. Prof. Wu had been unwell for nearly a year. I knew that this would happen one day, but refused to believe it would really come to pass. A few days ago, some students and I were on a field trip in Tibet. Sitting in our jeep, I watched the mountains and roadside as we drove, and tried to identify the plants along the route. The mountain roads in Tibet seem to always make my students sleepy. I woke them up. I told them that I had heard this first when Prof. Wu was on a field trip to Tibet. During the trip, he always kept an eye on the surroundings outside the vehicle, observing plants and taking notes. At the end of each day, he would prepare a check-list of plants for the region through which he had travelled. Back home it was the middle of the night, and I was awake. My thoughts had turned to my relationship with Professor Wu (Fig. 1).

**Figure 1:**
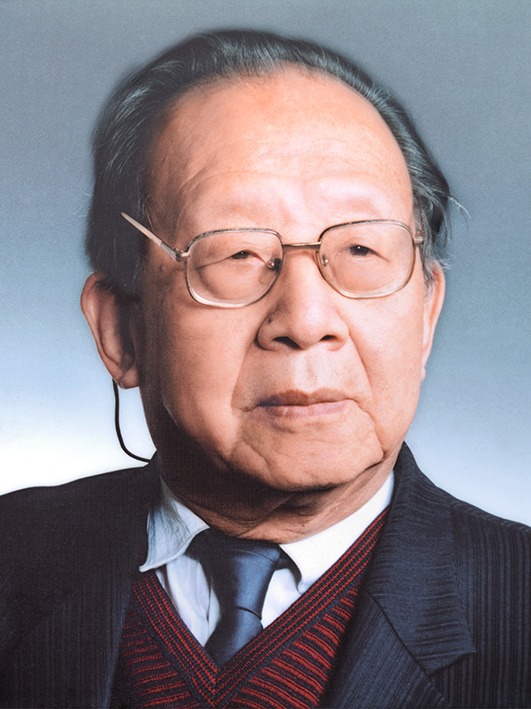
**Professor Zhengyi Wu (June 1916–June 2013)**

In 1986, one year after I had graduated with my Master’s degree from Nanjing Institute of Geology and Palaeontology, Chinese Academia Sciences, I, along with Dezhu Li and Jianqiang Li, became one of Professor Zhengyi Wu’s first PhD students (Fig. 2). Prof. Wu needed someone in his team to examine fossil plants for his research, and I was trained as a palaeobotanist. At the same time, I received a grant from the Chinese Academy of Sciences to pursue my doctorate overseas. I was very happy to have this opportunity but worried about how Prof. Wu would respond to the idea of me studying abroad. I was still a young graduate student, and did not know how to bring the subject up with my advisor. When Prof. Wu learned about my grant, however, he immediately encouraged me to study abroad. In the end, I was jointly supervised by Dr. Wilkinson from the Royal Botanical Gardens, Kew and Prof. Wu. I was at Kew for one year. I always felt honored to be one of Prof. Wu’s students.

**Figure 2:**
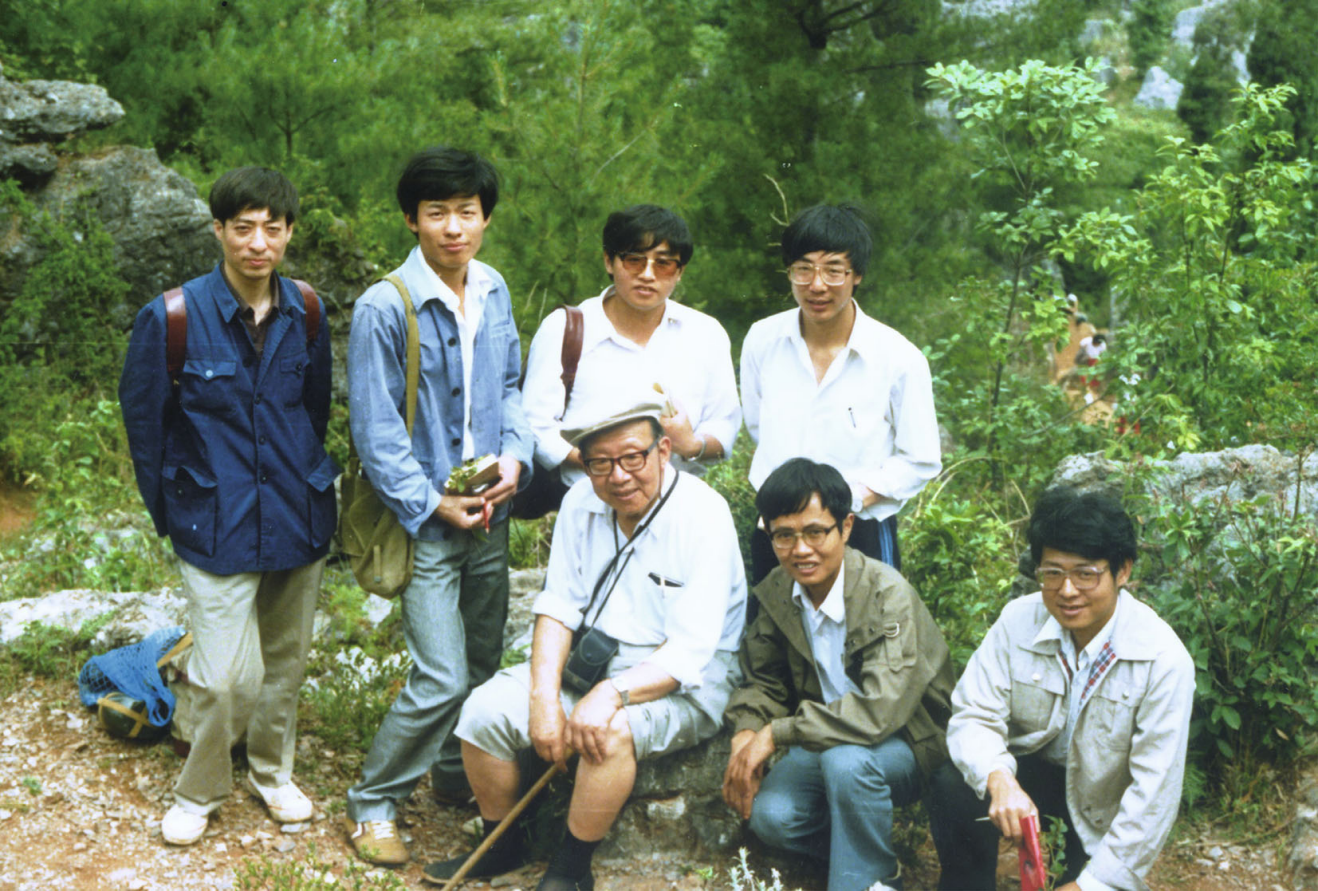
**Professor Zhengyi Wu and his students in 1988**. The first row from left: Zhengyi Wu, Zhekun Zhou, Hua Zhu; the second row from the left: Jianqiang Li, Dezhu Li, Qinger Yang, Yao Tang

I received my PhD in early 1991. At that time, Prof. Wu was leading a major project called “The Floristics of Chinese Seed Plants.” During the 1990s, this project was the largest of its kind at the National Natural Science Foundation of China. The project required a lot of fieldwork around China and many of my colleagues had already started to work in the field. According to Prof. Wu, the laboratories of floristic taxonomy, biogeography and ecology are in the field. Again and again, he told us, “As young students, you should work in the field, get your data first-hand in the field, and even contemplate the implications of what you find in the field.” With his encouragement, I joined a field trip to Motuo, SE Tibet, which is at the big bend of the gorge of Yalu Tsangpo in SE Tibet, one of the most remote places in China. There, I worked in the field for nine months. We collected more than 7,000 specimens from Motuo. Based on this work, we published over 20 papers, a book and received the National Prize for Natural Sciences from Chinese Academy of Sciences 1998. With the great vision of Prof. Wu, my own scientific career benefitted immensely.

In 1996, Prof. Wu, who was then already eighty years old, completed his project on “Floristics of Chinese Seed Plants”. But he was not content to rest. On the contrary, he said he still felt as if there were “four big mountains” on his shoulders. These “four big mountains” referred to books he was planning to write: *A Review of the Angiosperm Genera in China* (Wu et al., 2003a, b); *Florae Republicae Popularis Sinicae* (FRPS), volume one, general overview (Wu and Chen 2004); *The Areal-Types of Seed Plants and Their Origin and Differentiation* (Wu et al., 2006); *Floristics of Seed Plants from China* (Wu et al., 2010). In order to move these mountains off his shoulders, he asked four former students, Dr. Dezhu Li, Dr. Hang Sun, Dr. Hua Peng and me, for assistance. The plan was straightforward. Each student would help with one book. I was in charge of *The Areal-Types of Seed Plants and Their Origin and Differentiation*. The basic idea of this book originated from an article published in 1965 titled *The tropical floristic affinity of the Flora of China* (Wu, 1965). In this article, Prof. Wu analysed the distribution patterns of Chinese seed plants, classified them into 15 areal types and 31 sub-areal types, and then proposed the hypothesis of tropical affinity of Chinese floristics. He also proposed a hypothesis about the origin of East Asian floristics. Wu thought the regions of Indochina, South China and Southwest China, which have the richest primary families and genera, and have survived from the “Tertiary tropical”, are the core and cradle of East Asian floristics and have a tropical affinity (Wu, 1965). The classification of areal types of Chinese seed plants and the hypothesis that Chinese floristics have a tropical affinity were revised in the book *Chinese Physical Geography*—*Phytogeography* (*I*) (Wu and Wang, 1983). At the beginning of the project, I thought my job would be the easiest. The book had a well-developed framework and a lot of work had already been done. When we started preparing the manuscript, however, I found that Prof. Wu had compiled a substantial amount of new data. The content of the book had largely expanded. In the old book, he only provided an areal-type for Chinese genera of seed plants and a basic analysis of each areal-type. In the new book, Prof. Wu began with the basic concept of phytogeography. For instance, he addressed what floristic plant geography is, the difference between floristics and vegetation, and the research history of phytogeography. He continued by giving detailed explanations about the meaning of “areal”, and how different areal-types formed, particularly continental disjunction areal-types. He also addressed theories about dispersal and vicariance. Furthermore, he explained theories about the origin of disjunctions and proposed hypotheses on how continental drift impacted the formation of areal-types. In the third chapter, Prof. Wu employed his areal-type analysis method at the family level. In 2003, the part of this chapter that classifies 517 world families of seed plants into 18 areal types was published (Wu, 2003a, b). This scheme has been widely used in analysing the national and regional floras of China at various levels and is helpful in understanding biogeographic issues, such as endemism, vicariance and disjunctive distributions. In fact, this article is one of the most cited papers in China.

In this new book, Prof. Wu reviewed the taxonomic status of all 3,201 genera of Chinese seed plants and provided detailed information about the concept of every Chinese genus of seed plants in different systems and related references, synonyms, species number, detailed distribution information both in China and in the world and its areal-type. He summarised different systems of Gymnosperms, added distribution pattern data and proposed hypotheses for places of origin. This was a completely new book.

For me, helping to prepare the manuscript for this book was an incredible opportunity to learn from Prof. Wu. I had more time to discuss scientific issues with him (Fig. 3). I was honored when Prof. Wu added any of my comments or suggestions to the manuscript. I felt like I was working on a second PhD. It took eight years for Prof. Wu to finish the book. The manuscript contains over 10 million words and Prof. Wu, who by that time was ninety years old already, wrote down every single one (Fig. 4). He developed severe cataracts. His doctor advised him not to strain his eyes and to save his poor eyesight for his daily life. This did not make much of an impression on Prof. Wu. He continued to devote himself to his work.

**Figure 3:**
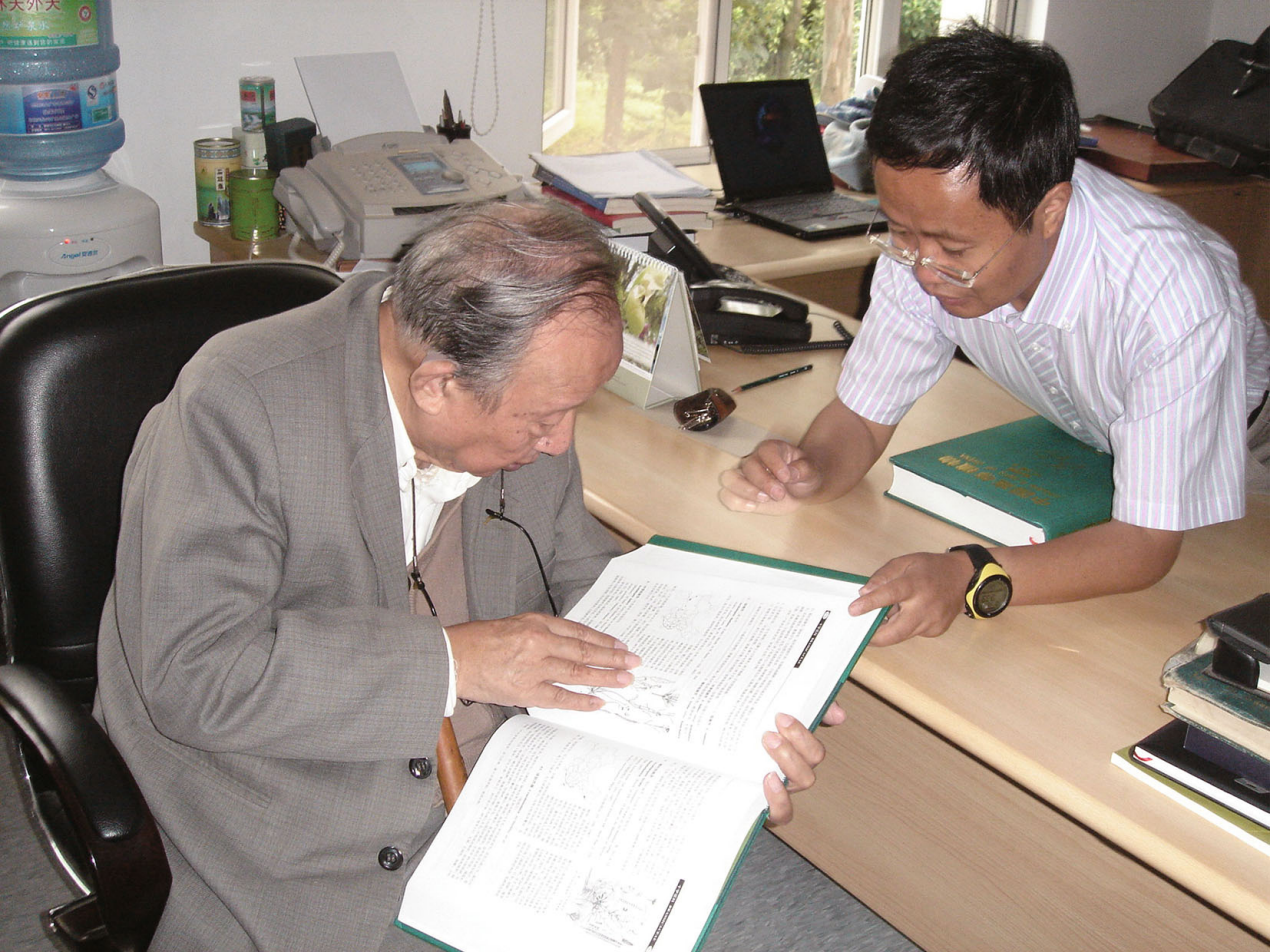
**Professor Zhengyi Wu and the author were discussing**

**Figure 4:**
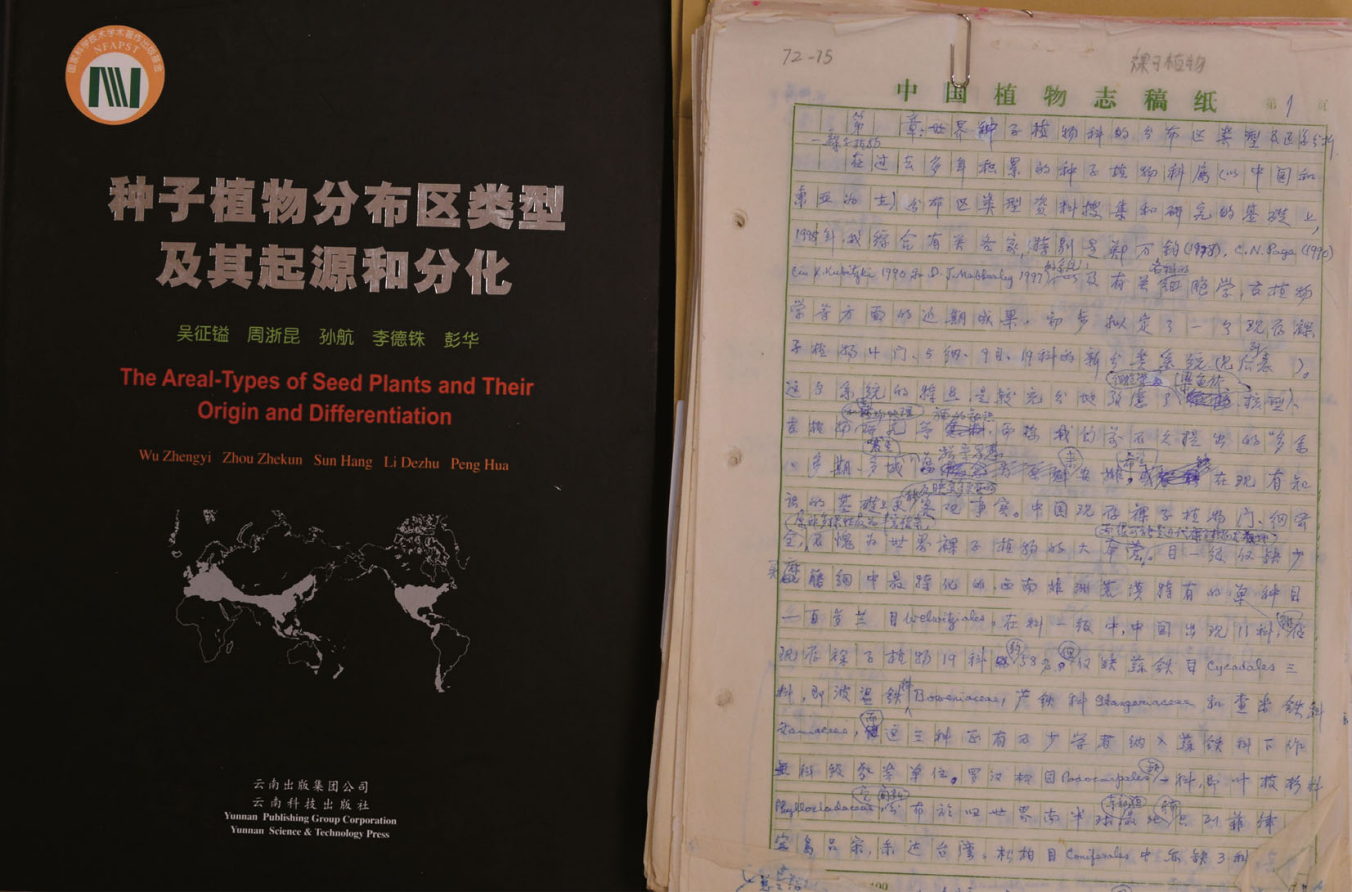
**The book and manuscript of Professor Zhengyi Wu**

The manuscript was finally completed, but I made one request. I asked Prof. Wu to write an autobiographical note for the book. He was happy to comply. One week later, Prof. Wu gave me his autobiographical note. When I read it, I was deeply moved. Prof. Wu was born in 1916. He developed an early interest in botany, inspired by his home garden and two books: *The Illustrated Book of Chinese Botany* (植物名实图考), written by Qing dynasty botanist Qiyong Wu, and *The Illustrated Book of Japanese Botany*. In middle school, he was fortunate to meet two Biology teachers: Mr. Shou Tang, his primary middle school Biology teacher, and Mr. Yao Tang, his middle school Biology teacher. They introduced him to fieldwork, and taught him to recognise plants and collect plant specimens. Profoundly inspired by his teachers, he decided to become a botanist. In 1933, he entered Tsinghua University in Beijing and received his BA in Biology four years later. By that time, the War of Resistance against Japanese Aggression had begun, and it was not possible to pursue research in botany. He moved, together with his University, to Kunming, Yunnan Province, which was home to the front during the war. He taught at National Southwestern Associated University and was a staff member at the Medicinal Plant Institute of the National Ministry of Education. During the war, teaching and research conditions were very poor. Regardless, Wu still tried his best to use his botanical knowledge to serve his country. Prof. Wu and other colleagues published a book called *ICONES Plantarum Medicarum e libro Tien-Nan-Pen-Tsao Lanmaoano Tom 1* (滇南本草图谱) (King et al., 1945). They investigated medicinal plants from an ancient Chinese medicinal plant book called *Tien-Nan-Pen-Tsao* (滇南本草). At the time, medicine was badly needed but in extremely short supply. Their research, which identified medicinal plants around Kunming, was able to help. Twenty-six medicinal plants, including a new genus, *Psammosilene*, W. C. Wu et C. Y. Wu were described in the book, which included hand-drawn illustrations. Everything required for publication—writing, drawing and printing—all had to be done by themselves.

Since then, Prof. Wu started his scientific career which spanned over 70 years. Many articles have recorded his contributions to botanical studies already, here I only want to emphasise his contributions in plant taxonomy and phytogeography. Prof. Wu became the fourth editor in chief of the Editorial Committee of *Florae Republicae Popularis Sinicae* (FRPS) in 1987, and 82 books made up of 54 volumes were published under his editorship (80 volumes in total). During the compilation of FRPS, he also chief edited another two great works: *Flora Yunnanica* and *Flora Xizangica*. In order that FRPS could be understood by non-Chinese speakers, Prof. Wu and Peter H. Raven became the co-chairs of the joint editorial committee. All 25 volumes of *Flora of China* were published in 2014, one year later Prof. Wu passed away unfortunately (Zhou and Sun, 2016).

By compiling the FRPS Prof. Wu and his colleagues answered the big question of how many plant species we have in China. His phytogeographcial research answered the next big question: “where are these plants growing”. His contribution to phytogeography has been recorded in the above-mentioned book *The Areal-Types of Seed Plants and Their Origin and Differentiation*. He proposed about origin and differentiation of distribution pattern of many seed plants from China. This hypothesis became a big legacy to biogeograpical research. Many research projects were proposed based on Prof. Wu’s hypothesis (Zhou and Sun, 2016).

We hope he will be remembered in the history of botany forever.

## References

[CR1] Wu ZY, Zhou ZK, Li DZ, Peng H, Sun H (2003). The areal types of the world families of seed plants. Acta Bot Yunnanica.

[CR2] King LP, Wu CY, Kuang KZ, Tsai TW (1945) ICONES PLANTARUM MEDICARUM e libro Tien-Nan-Pen-Tsao Lanmaoano. Tom.1., 25 spp., 26 plates

[CR3] Wu ZY, Lu AM, Tang YC, Chen ZD, Li DZ (2003). The families and genera of Angiosperms in China—a comprehensive analysis.

[CR4] Wu ZY, Chen XQ (2004). Florae Reipublicae Popularis Sinicae.

[CR5] Wu ZY, Zhou ZK, Sun H, Li DZ, Peng H (2006). The areal-types of seed plants and their origin and differentiation.

[CR6] Wu ZY, Sun H, Zhou ZK, Li DZ, Peng H (2010). Floristics of seed plants from China.

[CR7] Wu ZY, Wang HS (1983). Chinese physical geography—phytogeography (I).

[CR8] Zhou ZK, Sun H (2016). Wu Zhengyi and his contributions to plant taxonomy and phytogeography. Plant Diversity.

[CR9] Wu ZY (1965) The tropical floristic affinity of the Flora of China. Chinese Sci Bull 1:25–33 [吴征镒 (1965) 中国植物区系的热带亲缘. 科学通报 1:25–33.]

